# A New Approach of Modelling Bottom Edge Cutting in 4-Axis Rough Milling of Complex Parts and Its Application on Feed Rate Optimization

**DOI:** 10.3390/mi13122071

**Published:** 2022-11-25

**Authors:** Jie Zhao, Zhiyong Chang

**Affiliations:** 1 School of Mechanical Engineering, Northwestern Polytechnical University, Xi’an 710072, China; 2 Institute for Aero-Engine Smart Assembly of Shaanxi Province, Xi’an 710072, China

**Keywords:** bottom edge cutting, feed rate optimization, 4-axis rough machining, complex parts, blisk

## Abstract

Complex mechanical parts such as a blisk of aero-engines are commonly used in aerospace industry. These parts are complex in shape and their rough machining are conducted in 4-axis machine tools with end mills. The end mills are fully engaged into the workpiece material to be removed. Because of the complex cutter motion in 4-axis milling, the bottom edges of the end mills are involved in cutting with high possibility, resulting in an undesirable increase of cutting forces, tool deflection, and quick tool wear. To address this technical challenge, an analytical method is proposed to identify and evaluate the bottom edge cutting in 4-axis milling in this work. The motion of the cutter’s tool tip with respect to the workpiece is analyzed and the equations are formulated based on a basic interpolation algorithm. An approach to identifying and evaluating the bottom edge cutting is proposed. The increment of the cutting forces caused by the bottom edge cutting is taken into consideration to precisely evaluate the overall cutting forces. A feed rate optimization model is then established to control the cutting forces. The simulation and the experiment of rough milling of a blisk verify that the bottom edge cutting can be identified and the cutting force can be controlled by optimizing the feed rates without losing much machining efficiency.

## 1. Introduction

**Figure 1:**
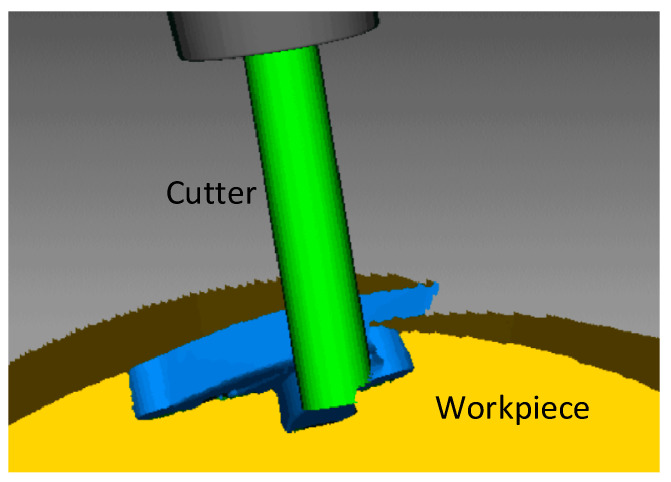
A flat end mill is fully engaged into the material of the workpiece in the rough milling of a blisk.

Complex mechanical parts such as blisks of aero-engines are commonly used in aeronautic and astronautic industries. These parts are complex in shape and their rough machining is conducted in 4-axis Computer Numerically Controlled (CNC) machine tools usually with end mills. The end mills are fully engaged into the workpiece material to be removed (see Figure 1). Because of the complex cutter motion in 4-axis milling, the bottom edges of the end mills are fully engaged in cutting with high possibility, resulting in an undesirable increase of cutting forces, tool deflection, and quick tool wear, even tool breakage. Therefore, it is crucial to identify and formulate the bottom edge cutting, and to reduce this undesirable effect during milling.

The bottom edges of cutters engage in cutting in many machining processes such as orbital drilling and plunge milling. Kong et al. [1] pointed out that the cutting edge on the bottom of the tool is the main cause of the cutting force in orbital drilling. Tian et al. [2] established a mathematical model to simulate the cutting depths and volume of the bottom cutting edges on the helical milling process. They found that the undeformed chip geometry is affected significantly and can be optimized by helical milling parameters to obtain a good cutting condition. Francesco et al. [3] developed a new approach to measure and compute the cutting force coefficients for end mills used in plunge milling. The method is important to predict the chatter conditions. Fredj et al. [4] found that augmentation of the chip cross-section is the cause of the increase of the cutting forces in deep plunge milling of titanium workpieces, and they set up a geometrical model to predict the enlargement of the chip cross-section.

However, the bottom edge cutting in rough milling with multi-axis machine tools has been more and more in the focus in the recent years. Zhu et al. [5] argued that when the lead angle of the tool axis is negative, the mechanistic model will lose accuracy if the bottom edge cutting effect is neglected. An improved mechanistic model of five-axis machining with a flat end mill was proposed. Wan et al. [6,7,8] proved that, by carefully calibrated cutting force coefficients and extended experiments, the influence of the bottom edge cutting is not negligible if the axial cutting depth is relatively small. Their work is limited to two-dimensional milling. Because of the additional bottom edge-induced cutting forces, the conservative machining parameters are usually adopted to avoid undesirable machining defects, causing low machining efficiency [9].

During the rough milling of complex parts, cutting parameters need to be carefully determined to achieve the objectives of rough machining. The cutting parameters include feed rate, spindle speed, width and depth of cut, etc., and the objectives could be different such as minimum machining time, maximum material removal rate, and maximum uniformity of the remaining volume at the end of roughing [10]. The cutting forces, moreover, need to be controlled to protect cutters and maintain stable machining. Many optimization methodologies are used in CNC machining to offer optimal process parameters [11]. Since the reduction in feed rate is an effective method to control cutting forces, Li et al. [12] divided a tool path into segments, then a heuristic method was employed to optimize the feed rate constrained by milling forces. Fu et al. [13] established a mapping relation between feed rate and cutting forces. The objective of optimizing the feed rate is to obtain better surface quality.

Unfortunately, the bottom edge cutting in 4-axis rough machining has not been successfully addressed in these existing research works. To address this technical challenge, an analytical method is proposed to identify and evaluate the bottom edge cutting in 4-axis milling in this paper. In Section 2, the motion of the cutter’s tool tip with respect to the workpiece is analyzed and formulated based on the basic interpolation algorithm, and a new approach to identify and evaluate the cutter’s bottom edge cutting is proposed. Section 3 establishes a feed rate optimization model. The increment of the cutting forces caused by the bottom edge cutting is taken into consideration to precisely evaluate the overall cutting forces. In Section 4, the simulation and the experiment of rough milling a blisk are rendered to verify this new approach.

## 2. Formula of Bottom Edge Cutting of Flat End Mills in 4-Axis Milling

In aerospace industry, the rough milling of complex parts is conducted in multi-axis machine tools, and the CNC controllers of the machine tools use different CNC interpolation algorithms. In order to evaluate the bottom edge cutting with high fidelity, the instantaneous cutter positions and orientations (or cutter locations) in machining should be accurately computed by using the actual machine kinematics and the interpolation algorithm of the CNC controller. Unfortunately, most conventional methods approximately calculate cutter locations without taking the interpolation algorithm into consideration, by which the large deviations from the actual locations are inevitable. This work adopts a 4-axis horizonal machine tool (e.g., X-, Y-, Z-, and B-axes) and a basic 4-axis CNC interpolation algorithm of constant-acceleration interpolation algorithm as example. The methodology of this work can be applied to 4-axis machines with different CNC controllers. The main steps of formulating bottom edge cutting are (1) according to two cutter locations in steps of an NC program, several instantaneous cutter locations in the machine coordinate system are sampled and calculated by using the constant-acceleration interpolation algorithm, (2) the instantaneous cutter locations are converted into the workpiece coordinate system by using the machine kinematics, and (3) the feed rate of the tool tip is formulated in the workpiece coordinate system, and it is adopted to evaluate the bottom edge cutting. The technical details are given as follows.

### 2.1. Representation of Instantaneous Cutter Locations Using the Basic Interpolation Algorithm

To cut the parts on 4-axis machine tool, discrete cutter locations of tool paths are preplanned using the CAM software, in which cutter positions (X-, Y-, and Z-coordinates) and orientations (B-coordinate) are calculated, and these Cutter Location Data (CLData) are translated to G-code feeding to the CNC controller. The feed rate for each cutter location is preplanned as well. In milling, the CNC controller interpolates many instantaneous cutter locations according to consecutive cutter locations in the NC program, and the cutter is controlled to move from one location to the next. Since the interpolation algorithm is not disclosed, it is reasonable to adopt a basic CNC interpolation algorithm, which is a constant-acceleration interpolation algorithm, in this work as an example. Although the adopted algorithm is different from those in other CNC controllers, this method is generic by using the actual algorithm.

The constant-acceleration interpolation algorithm is briefly described here. Suppose a step including two cutter locations $\left[\begin{array}{cccc} x_{i} & y_{i} & z_{i} & B_{i} \end{array}\right]$ and $\left[\begin{array}{cccc} x_{i+1} & y_{i+1} & z_{i+1} & B_{i+1} \end{array}\right]$ in the machine coordinate system are fed into the machine along with their feed rate $f_{i}$ and $f_{i+1}$. Then, the instantaneous cutter locations $\left[\begin{array}{cccc} x\left(t\right) & y\left(t\right) & z\left(t\right) & B\left(t\right) \end{array}\right]$ are interpolated with a constant-acceleration algorithm, where t is the time. The equation of the instantaneous cutter locations in the machine coordinate system is

(1) $$\left[\begin{array}{c} x\left(t\right)\\ y\left(t\right)\\ z\left(t\right)\\ B\left(t\right) \end{array}\right]=\left[\begin{array}{c} x_{i}+\frac{x_{i+1}-x_{i+1}}{L_{i}}\cdot \left(f_{i}\cdot t+\frac{a_{i}}{2}\cdot t^{2}\right)\\ y_{i}+\frac{y_{i+1}-y_{i+1}}{L_{i}}\cdot \left(f_{i}\cdot t+\frac{a_{i}}{2}\cdot t^{2}\right)\\ z_{i}+\frac{z_{i+1}-z_{i+1}}{L_{i}}\cdot \left(f_{i}\cdot t+\frac{a_{i}}{2}\cdot t^{2}\right)\\ B_{i}+w_{i}\cdot t \end{array}\right],t\in \left[\begin{array}{cc} 0, & \Delta_{i} \end{array}\right]$$

where $L_{i}$, $\Delta_{i}$, $a_{i}$, and $w_{i}$ represent the step length, the cutting time of this step, the average acceleration, and the average angular velocity, respectively. They are computed as

(2) $$\{\begin{array}{l} L_{i}=\sqrt{{\left(x_{i+1}-x_{i}\right)}^{2}+{\left(y_{i+1}-y_{i}\right)}^{2}+{\left(z_{i+1}-z_{i}\right)}^{2}}\\ \Delta_{i}=2\cdot \frac{L_{i}}{f_{i}+f_{i+1}}\\ a_{i}=\frac{f_{i+1}-f_{i}}{\Delta_{i}}\\ w_{i}=\frac{B_{i+1}-B_{i}}{\Delta_{i}} \end{array}$$

By using the machine kinematics, these cutter locations are converted into the workpiece coordinate system.

### 2.2. Kinematics of 4-Axis Machine Tool

**Figure 2:**
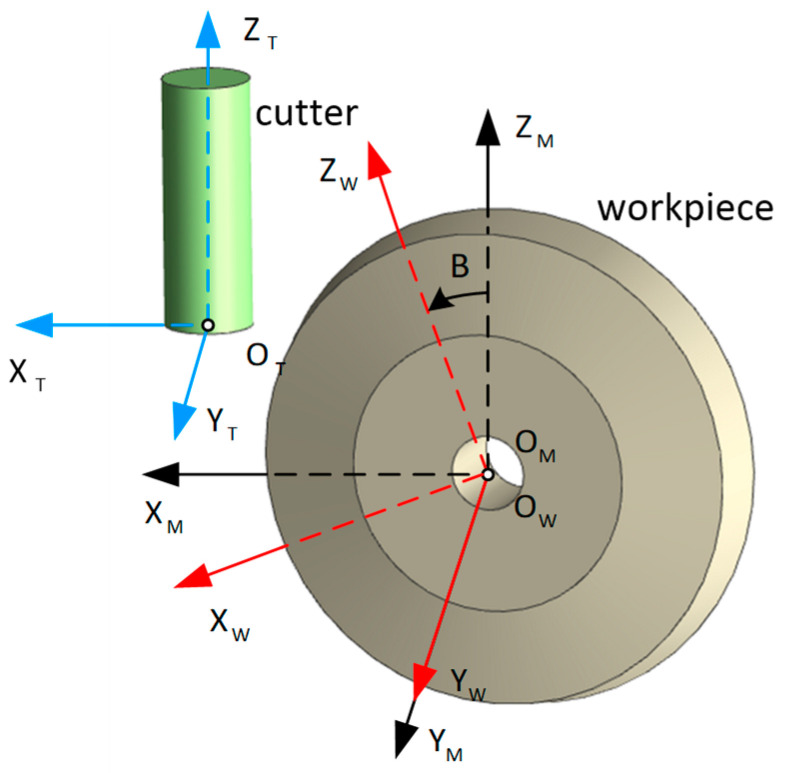
Three coordinate systems are defined.

The machine kinematics is established to convert the instantaneous cutter locations from the machine coordinate system into the workpiece coordinate system. Three coordinate systems are defined (see Figure 2). The origin $O_{M}$ of the machine coordinate system ${\mathrm{CS}}_{M}$ is located in the center of the workpiece, and its $X_{M}$-, $Y_{M}$-, and $Z_{M}$-axes are parallel to the x-, y-, and z-axes of the machine tool, respectively. The workpiece coordinate system ${\mathrm{CS}}_{W}$ is defined as: (a) ${\mathrm{CS}}_{W}$ coincides with the machine coordinate system ${\mathrm{CS}}_{M}$ when the rotation angle *B* of the machine tool’s table is zero; (b) ${\mathrm{CS}}_{W}$ is rotated around the $Y_{W}$-axis by angle *B* when the rotation angle *B* is not zero. In this case, angle *B* represents the tool orientation. At last, the tool coordinate system ${\mathrm{CS}}_{T}$ is defined by setting that (a) its origin $O_{T}$ is at the cutter’s tool tip and (b) its $X_{T}$-, $Y_{T}$-, and $Z_{T}$-axes are parallel to the s $X_{M}$-, $Y_{M}$-, and $Z_{M}$-axes of the machine coordinate system, respectively. The coordinates of the cutter’s tool tip in the machine coordinate system ${\mathrm{CS}}_{M}$ are $O_{T}={\left[\begin{array}{ccc} x\left(t\right) & y\left(t\right) & z\left(t\right) \end{array}\right]}^{T}$.

Based on these coordinate systems, the machine kinematics is established. The transformation matrix $M_{1}\left(t\right)$ from the tool coordinate system to the machine coordinate system is

$$M_{1}\left(t\right)=\left[\begin{array}{cccc} 1 & 0 & 0 & x\left(t\right)\\ 0 & 1 & 0 & y\left(t\right)\\ 0 & 0 & 1 & z\left(t\right)\\ 0 & 0 & 0 & 1 \end{array}\right]$$

and the transformation matrix $M_{2}\left(t\right)$ from the machine coordinate system to the workpiece coordinate system is

$$M_{2}\left(t\right)=\left[\begin{array}{cccc} \cos\left(B\left(t\right)\right) & 0 & -\sin\left(B\left(t\right)\right) & 0\\ 0 & 1 & 0 & 0\\ \sin\left(B\left(t\right)\right) & 0 & \cos\left(B\left(t\right)\right) & 0\\ 0 & 0 & 0 & 1 \end{array}\right]$$

The equivalent transformation matrix M(t) from the tool coordinate system to the workpiece coordinate system is

(3) $$\begin{array}{ll} M\left(t\right) & =M_{2}\left(t\right)\cdot M_{1}\left(t\right)\\ & =\left[\begin{array}{cccc} \cos\left(B\left(t\right)\right) & 0 & -\sin\left(B\left(t\right)\right) & x\left(t\right)\cdot \cos\left(B\left(t\right)\right)-z\left(t\right)\cdot \sin\left(B\left(t\right)\right)\\ 0 & 1 & 0 & y\left(t\right)\\ \sin\left(B\left(t\right)\right) & 0 & \cos\left(B\left(t\right)\right) & z\left(t\right)\cdot \cos\left(B\left(t\right)\right)+x\left(t\right)\cdot \sin\left(B\left(t\right)\right)\\ 0 & 0 & 0 & 1 \end{array}\right] \end{array}$$

### 2.3. Identification and Evaluation of Bottom Edge Cutting

**Figure 3:**
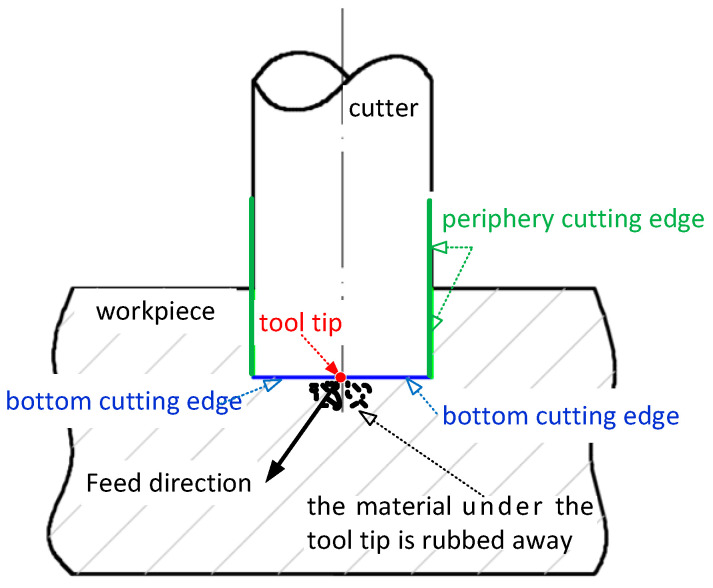
The material under the tool tip is rubbed away.

For a flat end mill, its periphery cutting edges always cut the workpiece material at a preplanned cutting speed (see Figure 3). The bottom cutting edges, however, cut the workpiece material at a lower cutting speed than the preplanned one. The closer the tool tip to the bottom cutting edge, the lower the cutting speed. The tool tip, which is the center of the bottom cutting edges and located on the cutter axis, has zero cutting speed. In effect, the workpiece material under the tool tip is rubbed away rather than cut off (see Figure 3), resulting in large cutting forces and quick tool wear. Therefore, the tool tip among the bottom edges is adopted to evaluate the bottom edge cutting.

In the workpiece coordinate system, the instantaneous cutter locations, which include the tool tip position P(t) and the orientation of the cutter axis A(t), are calculated as

(4) $$\left[\begin{array}{c} P\left(t\right)\\ 1 \end{array}\right]=M\left(t\right)\cdot \left[\begin{array}{c} 0\\ 0\\ 0\\ 1 \end{array}\right]=\left[\begin{array}{c} x\left(t\right)\cdot \cos\left(B\left(t\right)\right)-z\left(t\right)\cdot \sin\left(B\left(t\right)\right)\\ y\left(t\right)\\ z\left(t\right)\cdot \cos\left(B\left(t\right)\right)+x\left(t\right)\cdot \sin\left(B\left(t\right)\right)\\ 1 \end{array}\right]$$

and

(5) $$\left[\begin{array}{c} A\left(t\right)\\ 0 \end{array}\right]=M\left(t\right)\cdot \left[\begin{array}{c} 0\\ 0\\ 1\\ 0 \end{array}\right]=\left[\begin{array}{c} -\sin\left(B\left(t\right)\right)\\ 0\\ \cos\left(B\left(t\right)\right)\\ 0 \end{array}\right]$$

Due to the complex motion in 4-axis machining, the feed directions of the tool tip at the instantaneous cutter locations are different. The feed direction V(t) is formulated by

(6) $$\begin{array}{ll} V\left(t\right) & =\frac{dP\left(t\right)}{dt}\\ & =\left[\begin{array}{c} \frac{dx\left(t\right)}{dt}\cdot \cos\left(B\left(t\right)\right)-\omega_{i}\cdot x\left(t\right)\cdot \sin\left(B\left(t\right)\right)-\frac{dz\left(t\right)}{dt}\cdot \sin\left(B\left(t\right)\right)-w_{i}\cdot z\left(t\right)\cdot \cos\left(B\left(t\right)\right)\\ \frac{y_{i+1}-y_{i}}{L_{i}}\cdot \left(f_{i}+a_{i}\cdot t\right)\\ \frac{dz\left(t\right)}{dt}\cdot \cos\left(B\left(t\right)\right)-w_{i}\cdot z\left(t\right)\cdot \sin\left(B\left(t\right)\right)+\frac{dx\left(t\right)}{dt}\cdot \sin\left(B\left(t\right)\right)+w_{i}\cdot x\left(t\right)\cdot \cos\left(B\left(t\right)\right) \end{array}\right] \end{array}$$

where $\frac{dx\left(t\right)}{dt}$ and $\frac{dz\left(t\right)}{dt}$ are the derivatives of x(t) and z(t) in terms of time t, and they are calculated by

(7) $$\{\begin{array}{c} \frac{dx\left(t\right)}{dt}=\frac{x_{i+1}-x_{i}}{L_{i}}\cdot \left(f_{i}+a_{i}\cdot t\right)\\ \frac{dz\left(t\right)}{dt}=\frac{z_{i+1}-z_{i}}{L_{i}}\cdot \left(f_{i}+a_{i}\cdot t\right) \end{array}$$

**Figure 4:**
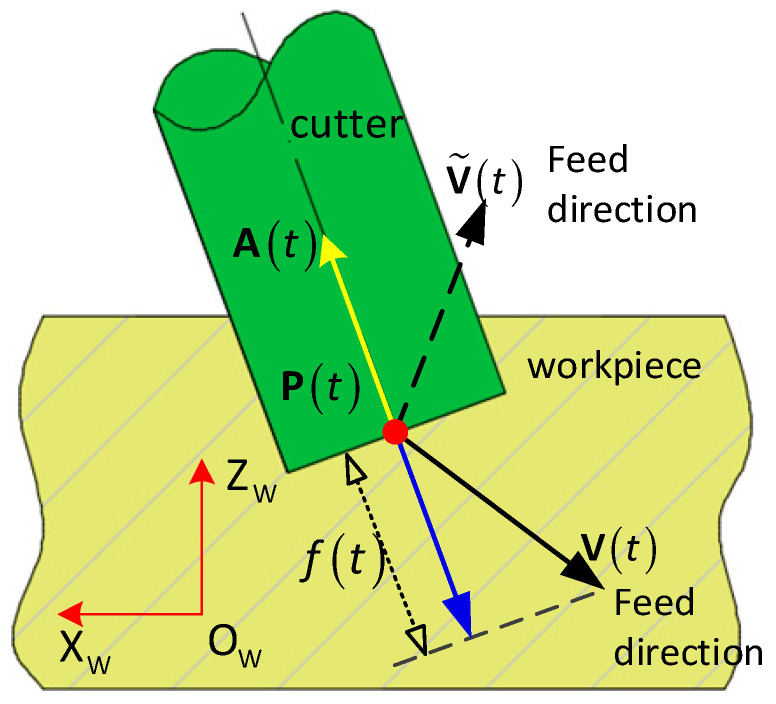
The feed direction of the tool tip is either leaving out of or heading into the workpiece material under the tool tip.

At some moments, the feed direction $\tilde{V}\left(t\right)$ of the tool tip points inside the cutter (see Figure 4), in which $A\left(t\right)\cdot \tilde{V}\left(t\right)>0$ holds. Since the tool tip is moving toward the inside of the cutter and leaving the workpiece material under the tool tip, the tool tip is not involved in cutting. While at some particular moments, the feed direction V(t) of the tool tip points out of the cutter, in which A(t)·V(t)<0 holds. The tool tip is moving toward the outside of the cutter and heading into the workpiece material under the tooltip, the tool tip is thus involved in cutting. Furthermore, the actual instantaneous feed rate f(t) of the tool tip is the signed length of projection vector of V(t) onto the opposite of A(t), and it is computed by

(8) $$f\left(t\right)=-A\left(t\right)\cdot V\left(t\right)=-w_{i}\cdot \left[x_{i}+\frac{x_{i+1}-x_{i}}{L_{i}}\cdot \left(f_{i}\cdot t+\frac{a_{i}}{2}\cdot t^{2}\right)\right]-\frac{z_{i+1}-z_{i}}{L_{i}}\cdot \left(f_{i}+a_{i}\cdot t\right)$$

**Figure 5:**
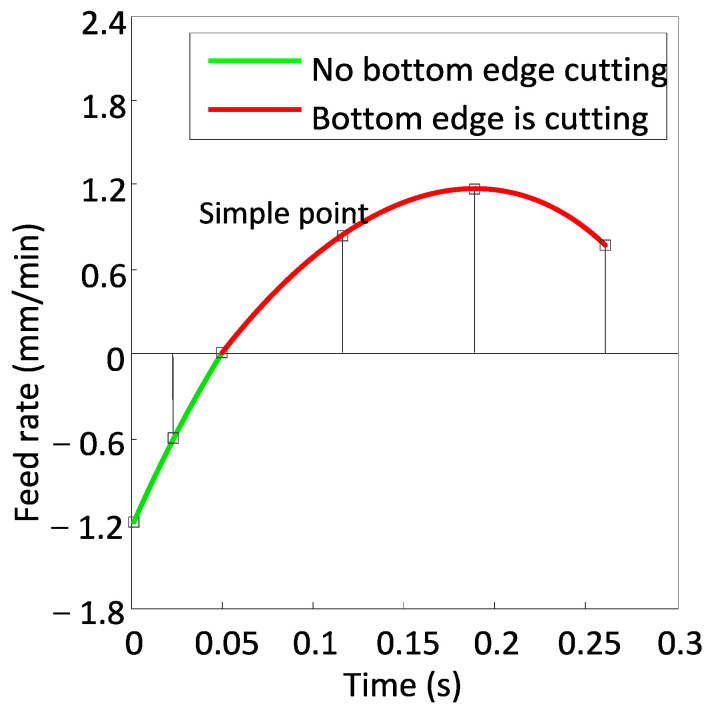
A number of instantaneous feed rates of the tool tip on a step are sampled and the maximum instantaneous bottom edge feed rate is determined.

From Equation (8), a positive feed rate f(t) indicates the bottom edge cutting, while the negative f(t) suggests no bottom edge cutting. An example of the instantaneous feed rate of the tool tip in a step is shown in Figure 5. From the beginning of cutting to the moment of 0.05 s, no bottom edge cutting occurs (green curve in Figure 5). After that moment, the bottom edge is involved in cutting (red curve in Figure 5). 

To evaluate the bottom edge cutting, a number of instantaneous feed rates of the tool tip on a step are sampled. The maximum of them is called the maximum instantaneous bottom edge feed rate on the step, and it is denoted as $f_{i}^{M}$ (see Figure 5). The bottom edge cutting occurs if $f_{i}^{M}$ is positive. The larger the $f_{i}^{M}$, the severer the bottom edge cutting. 

## 3. Feed Rate Optimization Model Considering Bottom Edge Cutting

The process of milling is unstable if the cutting forces are larger than the normal, resulting in chatters, quick tool wear or cutting-edge chipping. In the worst case, the cutter breaks. In the end of the last section, we have theoretically demonstrated that the bottom edge may be engaged in cutting and it is represented by the maximum instantaneous bottom edge federate computed with Equation (8). Because the bottom edge cutting increases the cutting forces, the effect of bottom edge cutting is taking into consideration to control the cutting forces, achieving stable cutting. 

In this research study, an optimization model is constructed to control the resultant cutting forces. The objective of the optimization is to minimize the cutting time because the purpose of rough milling is to remove the large amount of material of billets as quickly as possible. To identify the optimization variables, a few key factors of the rough milling process are analyzed. The tool paths are workpiece geometry-dependent and the spindle speed is determined by the workpiece material and the cutters; these two factors are not modified in machining. Therefore, the feed rates are selected as optimization variables. Each cutter location in a tool path can have its own feed rate. For a step, if the maximum instantaneous bottom edge feed rate $f_{i}^{M}$ is positive, the feed rates on the cutter locations of this step are marked as to-be-optimized. All the to-be-optimized feed rates in a tool path are found and denoted as $f_{j}^{O},j=1,2,\cdots ,m$. The optimization model is formulated as

(9) $$\mathrm{minimize}\;T\left(f_{1}^{O},f_{2}^{O},\cdots ,f_{m}^{O}\right)$$

where T is total cutting time and $\;T\left(f_{1}^{O},f_{2}^{O},\cdots ,f_{m}^{O}\right)=\sum_{i}^{n}\Delta_{i}$, $\Delta_{i}$ is the cutting time of a step and computed with Equation (2). n represents the number of steps in the tool path. The optimization model is subject to the following constraints.

**Constraint 1**

The cutting forces must be controlled within an acceptable range. Here, in this work, a practical and simple control method is proposed. Based on the well-established mechanistic model of cutting force, milling forces are proportional to the area of the swept cross-section of the cutting edges. For the periphery cutting edges, the area $A_{i}^{P}$ is calculated by $A_{i}^{P}=a_{p}\cdot f_{i}^{z}$, where $a_{p}$ is the axial cutting depth and $f_{i}^{z}$ is the feed rate per tooth. $f_{i}^{z}=\frac{f_{i}}{S\cdot Z}$, where S and Z are spindle speed and cutter’s tooth number, respectively. $f_{i}$ is the preplanned feed rate. The cutting force caused by the periphery cutting edges $F_{i}^{P}$ is computed with

(10) $$F_{i}^{P}=C_{P}^{}\cdot A_{i}^{P}=\frac{C_{P}^{}\cdot a_{p}\cdot f_{i}}{S\cdot Z}$$

where $C_{S}^{}$ is the cutting force coefficient of the side edge of the cutter. When the bottom edges are engaged in cutting, the cutting force coming from the bottom edge is calculated by $F_{i}^{B}=C_{B}^{}\cdot A_{i}^{B}$,where $C_{B}^{}$ is the cutting force coefficient of the bottom cutting edge. $A_{i}^{B}=r\cdot f_{i}^{M}$, where r represents the cutter radius and $f_{i}^{M}$ is the maximum instantaneous bottom edge feed rate. It is worth noting that $f_{i}^{M}\left(f_{i}\right)$ varies according with the feed rate $f_{i}$. According to Zhu’s research [5], $C_{B}^{}$ is fairly close to $C_{P}^{}$, thus we approximately assume $C_{B}^{}=C_{P}^{}$ in our research. Therefore, $F_{i}^{B}$ is computed with

(11) $$F_{i}^{B}=C_{B}^{}\cdot A_{i}^{B}=\frac{C_{P}^{}\cdot r\cdot f_{i}^{M}\left(f_{i}\right)}{S\cdot Z}$$

After optimization, the feed rate $f_{i}$ is replaced by the optimized one $f_{i}^{O}$, the resultant cutting force combining the periphery cutting edge and the bottom cutting edge is

(12) $$F_{i}^{O}=\frac{C_{P}^{}\cdot \left(a_{p}\cdot f_{i}^{O}+r\cdot f_{i}^{M}\left(f_{i}^{O}\right)\right)}{S\cdot Z}$$

To eliminate the additional effect of the bottom edge cutting on the resultant cutting force, the cutting force caused only by the periphery cutting edges $F_{i}^{P}$ is taken as the acceptable threshold. This threshold requires $F_{i}^{O}<F_{i}^{P}$. By plugging Equation (10) and Equation (12) into the inequation and simplifying it, the first constraint is

(13) $$f_{i}^{O}<f_{i}-\frac{r}{a_{p}}\cdot f_{i}^{M}\left(f_{i}^{O}\right)$$

**Constraint 2**

To ensure that the cutter’s acceleration does not exceed the dynamics of the machine tools, the second constraint is

(14) $$a_{i}<a_{\max}$$

where $a_{\max}$ is the limit of acceleration on the linear axis. The limit of angular acceleration is not handled in this study because the constant-acceleration interpolation algorithm adopted does not take the angular acceleration into consideration.

## 4. Verification and Application

**Figure 6:**
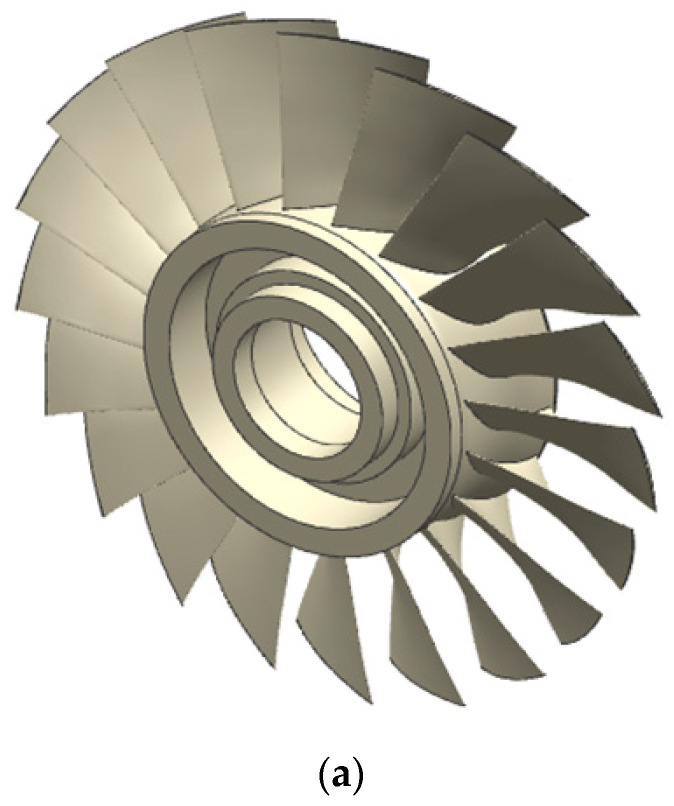

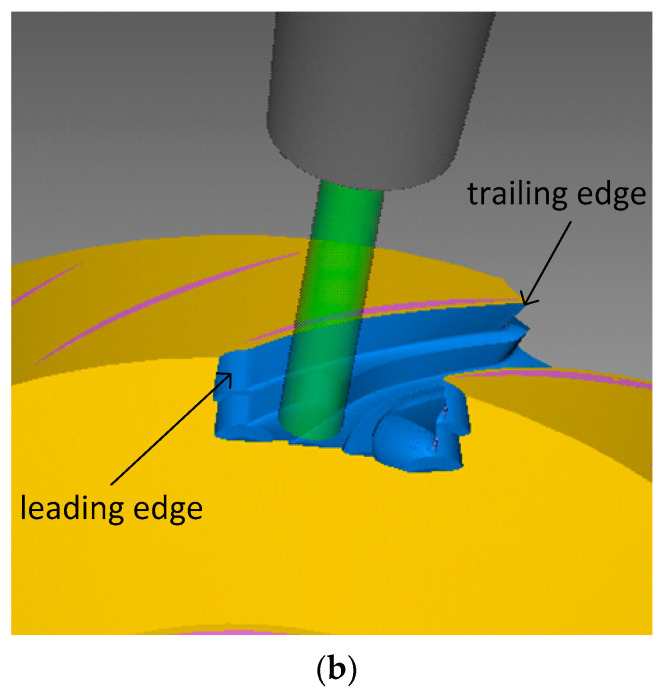
(**a**) A blisk with 20 channels, and (**b**) the cutter cuts the channel from the leading edge to the trailing edge.

To demonstrate its validity, this approach is applied to a tool path of rough machining a blisk in a 4-axis machine tool. Twenty channels of the blisk need to be machined (see Figure 6a). Each channel is 43 mm in height and 42 mm in width. The channel is machined with a flat end mill of 5 mm radius. Twenty-six tool paths are planned using UG NX software, and the channel is cut layer by layer. The axial cutting depth $a_{p}$ is 4 mm. One of the tool paths is shown in Figure 6b. This tool path cuts the channel from the leading edge to the trailing edge. The preplanned feed rates are determined based on tool vendor’s recommendations and cutting experiments.

**Figure 7:**
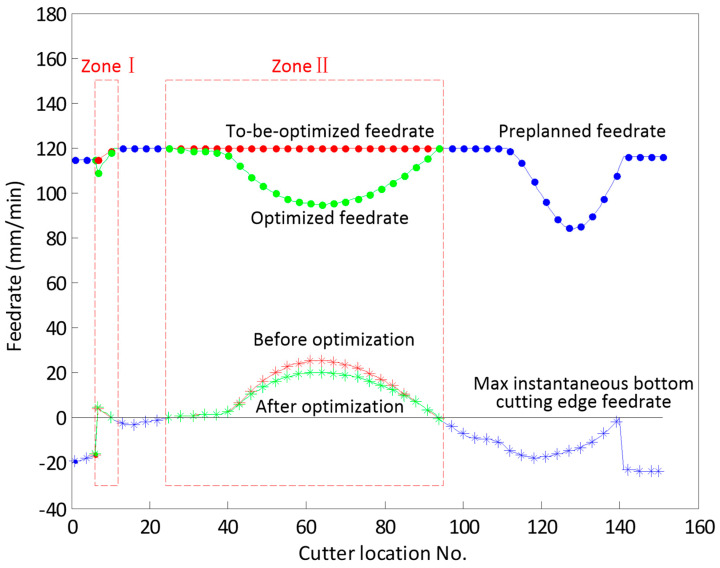
The bottom edge cutting is identified and the feed rates are optimized.

According to the preplanned tool path and its feed rate, the maximum instantaneous bottom edge feed rates are computed by using the evaluation method proposed in Section 2, and they are plotted with a red asterisk line in Figure 7. It is clear that the bottom edge is involved in cutting in zone I (Cutter location no. 7 to 11) and zone II (Cutter location no. 25 to 94). Therefore, these preplanned feed rates, from $f_{7}$ to $f_{11}$ and from $f_{25}$ to $f_{94}$, are marked as to-be-optimized. They are plotted with a red dotted line in Figure 7. 

The optimization model is established as described in Section 3, in which the maximum acceleration $a_{\max}$ of the machine tool is set to 9.8 $m/s^{2}$. The Genetic Algorithm (GA) built in MATLAB is employed to solve the optimization model. Since this paper does not focus on the optimization method, GA parameters with MATLAB default values are adopted, except that the size of population is set to twice the number of cutter locations marked to-be-optimized. The optimized feed rates are determined and plotted with a green dotted line in Figure 7. Accordingly, the maximum instantaneous bottom edge feed rates determined by these optimized feed rates are re-evaluated and plotted with a green asterisk line. In the zone near the cutter location no. 63, the bottom edge cutting reaches its maximum, and the optimized feed rate is significantly reduced from 120 mm/min to 95 mm/min. Therefore, the cutting force could be controlled and the tool is protected. After optimization, the machining time is slightly increased from 30.6 s to 32.4 s. It is readily seen that this approach can effectively decrease the effect of bottom edge cutting by optimizing the feed rate.

**Figure 8:**
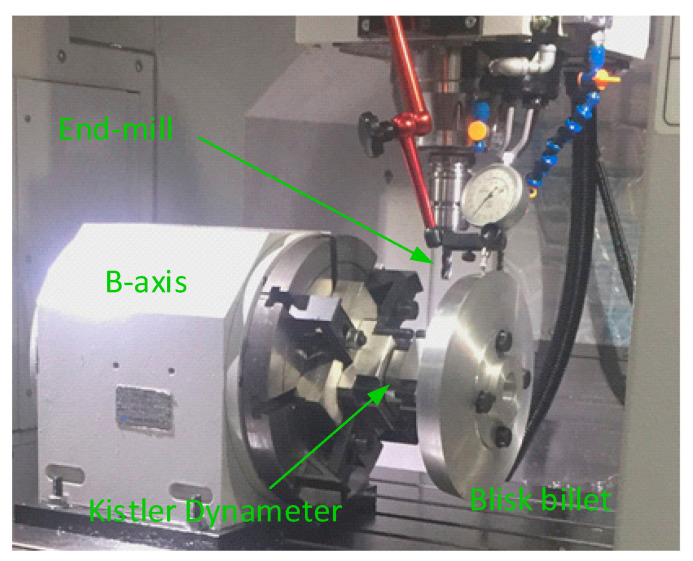
A Kistler dynameter is set up to measure the cutting forces.

A further experiment is conducted to verify the reduction in cutting force. A tool path is programed using UG NX to cut the channel. In the experiment, a dynameter of Kistler 9367C is employed to measure the cutting forces before and after the optimization (see Figure 8).

**Figure 9:**
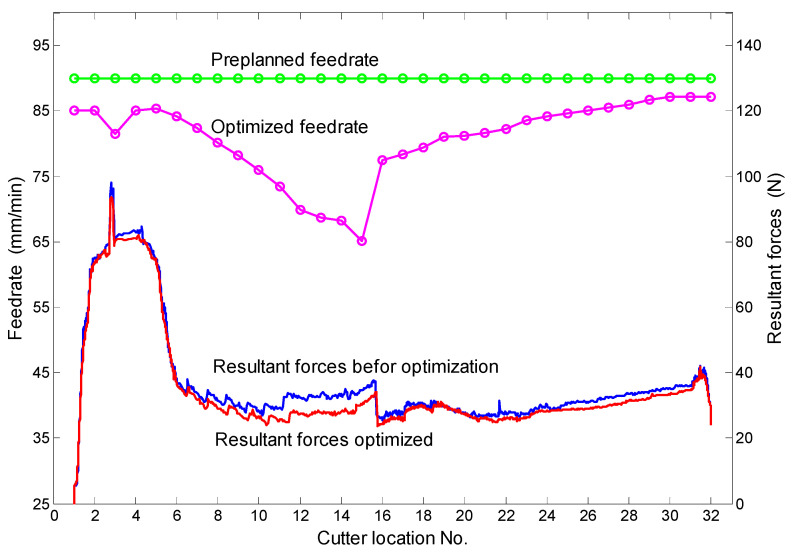
Comparisons of the feed rates and the cutting forces before and after optimization.

By evaluating the bottom edge cutting, the feed rates all over the tool path are marked as to-be-optimized. The preplanned and the optimized feed rates are plotted in Figure 9. With the preplanned and the optimized feed rates, two channels are cut in the 4-axis machine tool and their cutting forces are measured, respectively. The resultant forces are also plotted in Figure 9. 

As can be seen in Figure 9, the cutting forces vary significantly along the whole path due to the complex cutter motion in 4-axis machining. Moreover, careful observation of the cutting forces indicates that the optimized cutting forces are reduced more or less, according to the reduction in the feed rate. In the area near the cutter location no. 14, the cutting forces are reduced apparently, since the feed rate drops down dramatically about 25% in the area. The maximum reduction in cutting forces is on the cutter location no. 11, the cutting force is reduced by 28.7% (from 35.2 N to 25.1 N) when the feed rate dropped down by 19.3% (from 90 mm/min to 72.6 mm/min). The actual machining time is increased from 31.5 s before optimization to 34.8 s after optimization.

## 5. Conclusions

This paper proposes an analytical approach to identify and evaluate the bottom edge cutting in the rough milling of complex parts. By using the CNC interpolation algorithm, the motion of the cutter’s tool tip with respect to the workpiece material is formulated. As the benefit of doing so, the motion of the bottom edges of the cutters are represented precisely in accordance with the specific CNC controllers. The mechanism of the bottom edge cutting is analyzed. The motion vector of the tool tip is projected onto the opposite of the cutter’s axis to calculate the signed feed rate of the tool tip engaging into the workpiece material. A number of the feed rates are sampled within the step of tool paths and the maximum of these feed rates is used to evaluate the bottom edge cutting. The sign of this feed rate is employed to identify the bottom edge cutting. Then, the cutting forces caused by bottom edges are estimated by computing the area of the cross-section swept by the bottom edges multiped by the cutting force coefficient. An optimization model is established to achieve the high roughness efficiency by optimizing the feed rates, as well as constraining the combination of bottom edge cutting forces and the periphery edge cutting forces. 

The results of simulation and experiment show that the bottom edge cutting is identified and the cutting force is controlled by optimizing the feed rates without losing much efficiency in rough milling blisks of aero-engines. We believe that this approach can be directly implemented in rough milling impellers of aero-engines, and other complex parts in industry.

## Data Availability

Not applicable.
